# Investigation of Hippo pathway-related prognostic lncRNAs and molecular subtypes in liver hepatocellular carcinoma

**DOI:** 10.1038/s41598-023-31754-x

**Published:** 2023-03-20

**Authors:** Qiongfei Su, Fengyang Hua, Wanying Xiao, Baoqiu Liu, Dongxia Wang, Xintian Qin

**Affiliations:** 1 Department of Oncology, The First Affiliated Hospital of Guangdong, Pharmaceutical University, Guangzhou, China; 2 Department of Radiation Oncology, Affiliated Dongguan People’s Hospital, Southern Medical University, Dongguan, China

**Keywords:** Cancer, Genetics, Immunology, Molecular biology

## Abstract

This study aimed to investigate Hippo pathway-related prognostic long noncoding RNAs (lncRNAs) and their prognostic value in liver hepatocellular carcinoma (LIHC). Expression and clinical data regarding LIHC were acquired from The Cancer Genome Atlas and European Bioinformatics Institute array databases. Hippo pathway-related lncRNAs and their prognostic value were revealed, followed by molecular subtype investigations. Differences in survival, clinical characteristics, immune cell infiltration, and checkpoint expression between the subtypes were explored. LASSO regression was used to determine the most valuable prognostic lncRNAs, followed by the establishment of a prognostic model. Survival and differential expression analyses were conducted between two groups (high- and low-risk). A total of 313 Hippo pathway-related lncRNAs were identified from LIHC, of which 88 were associated with prognosis, and two molecular subtypes were identified based on their expression patterns. These two subtypes showed significant differences in overall survival, pathological stage and grade, vascular invasion, infiltration abundance of seven immune cells, and expression of several checkpoints, such as CTLA-4 and PD-1/L1 (*P* < 0.05). LASSO regression identified the six most valuable independent prognostic lncRNAs for establishing a prognosis risk model. Risk scores calculated by the risk model assigned patients into two risk groups with an AUC of 0.913 and 0.731, respectively, indicating that the high-risk group had poor survival. The risk score had an independent prognostic value with an HR of 2.198. In total, 3007 genes were dysregulated between the two risk groups, and the expression of most genes was elevated in the high-risk group, involving the cell cycle and pathways in cancers. Hippo pathway-related lncRNAs could stratify patients for personalized treatment and predict the prognosis of patients with LIHC.

## Introduction

Hepatocellular carcinoma (LIHC) is the sixth most prevalent malignancy worldwide and the second most common cause of cancer-associated mortality^1^. Treatment strategies, including surgery and transplantation, can enhance the quality of life of patients with LIHC^2^. However, owing to the current limitations of relevant drugs and the inefficiency of early diagnosis, the survival rate of patients with LIHC is unsatisfactory^3^. Thus, a thorough investigation of the molecular mechanisms of carcinogenesis in LIHC is vital for identifying novel therapeutic options.

The pathological process of LIHC is extremely complex, involving the cascading effects of many signals, which ultimately leads to changes in key molecular outcomes in vivo and tumor progression^4^. As an evolutionarily conserved cascaded signaling network, Hippo pathway is implicated in various biological functions, such as cell proliferation and organ growth^5^. The Hippo pathway is reported to regulate the regeneration, development, and metabolism of the liver, and its perturbations result in liver diseases, including LIHC^6^. Long non-coding RNAs (lncRNAs) are a kind of non-coding RNAs, which have been demonstrated to participate the initiation and progression of various tumors by modulating biological behavior of tumor cells, especially in LIHC^7^. Interestingly, lncRNAs are found to interact with Hippo cascade, in which lncRNAs can regulate or be modulated by Hippo signaling pathway, and thereby promote the development of cancerous phenotypes^8^. A previous study indicated that the lncRNA HOTAIR triggers the Hippo pathway by directly binding to SAV1, which further contributes to the progression of renal cell carcinoma^9^. A recent study showed that the lncRNA NEAT1 could accelerate the self-renewal mechanism of LIHC cells via the PKA/Hippo signaling pathway^10^. In fact, the lack of proper biomarkers has hindered the prognosis and treatment stratification of patients with LIHC^11^. It has been proven that prognostic subtypes are a breakthrough for revealing potential clinical therapeutic strategies for liver cancer^12^. Unfortunately, the prognostic role and molecular mechanism of the Hippo pathway-related lncRNAs in LIHC progression remains unclear.

In recent years, the rapid development of sequencing technology and computational tools or bioinformatics analysis methods make it possible to investigate the involvement of lncRNAs in the progression of tumors^13–16^. For example, by analyzing sequencing data using various computational or bioinformatics analysis methods, Zhang et al.^13^ established a prognostic signature based on six Cuproptosis-related lncRNA to predict the response to immunotherapy for HCC patients. In the current study, molecular subtypes were investigated based on Hippo pathway-related prognostic lncRNAs using unsupervised cluster analysis, followed by a comparison of the differences between subtypes in survival, clinical characteristics, tumor-infiltrating immune cells, and immune checkpoints. Additionally, several Hippo pathway-related lncRNAs associated with prognosis were identified to develop a prognosis risk model, and the survival and molecular features of different risk groups were investigated.

## Material and methods

### Ethics statements

Information patients consent is not required for the use of our data from The Cancer Genome Atlas (TCGA) database and the European Bioinformatics Institute (EBI) array database. All methods comply with relevant guidelines and regulations.

### Data sources and preprocessing

**Figure 1:**
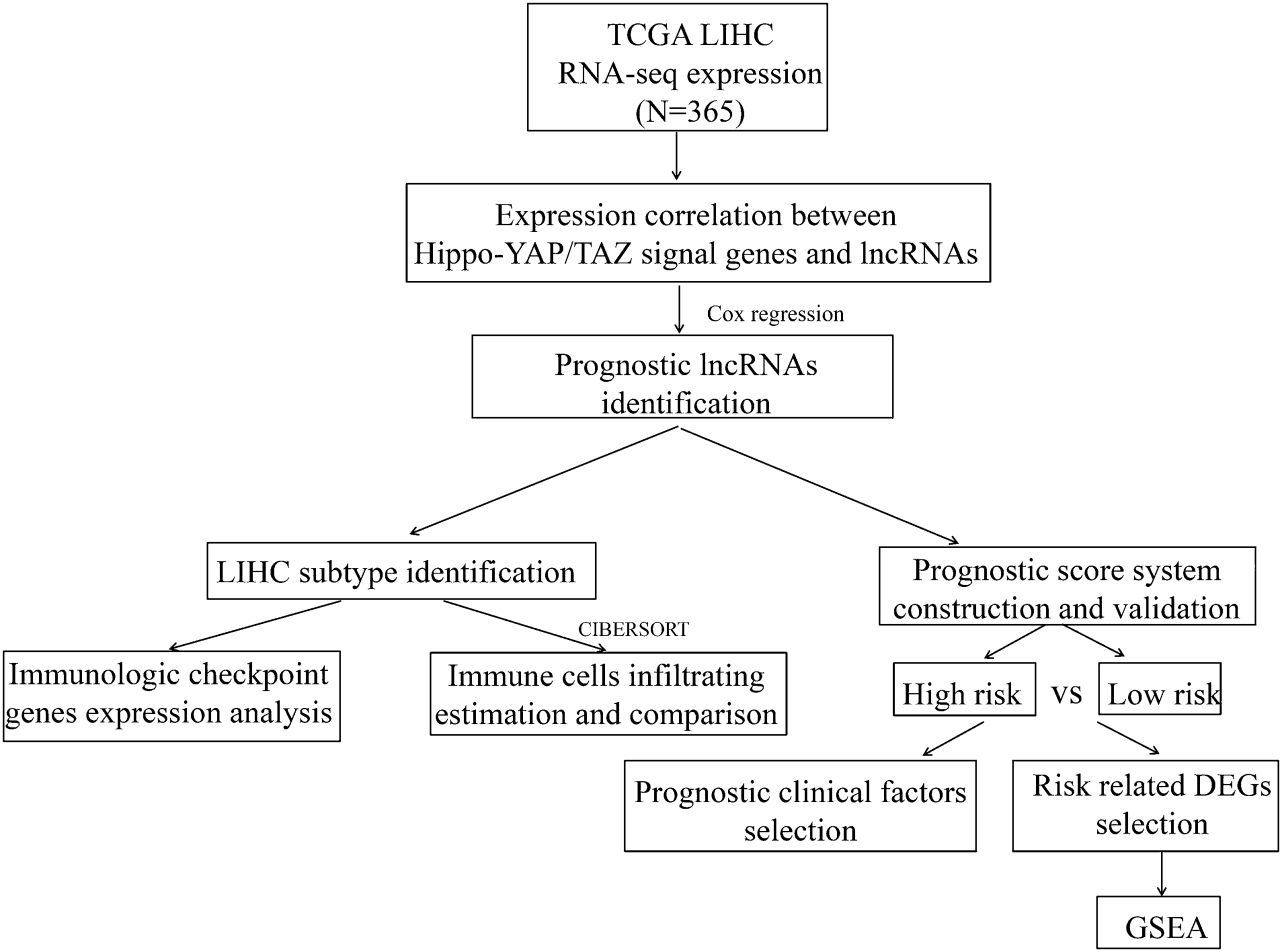
The workflow of this study.

The log2(FPKM+1) RNA-seq data of LIHC in The Cancer Genome Atlas (TCGA) database (https://www.cancer.gov/)^17^ were acquired for analysis, and 423 samples were obtained. Among which, 373 were tumor samples, and 365 tumor samples have clinical phenotype data. Consequently, these 365 samples were used as the training dataset in this study. The annotation of lncRNA and mRNA in this TCGA expression profile data was performed based on the transcript ID in the Illumina HiSeq 2000 RNA Sequencing platform. Additionally, E-TABM-36 dataset for LIHC in the European Bioinformatics Institute (EBI) array database (http://www.ebi.ac.uk/) was used as a validation dataset. This dataset contained 65 samples, and 44 samples of which had clinical survival data. Data in E-TABM-36 dataset was generated on platform of GPL96 Affymetrix GeneChip Human Genome HG-U133A, and were annotated based on the annotation files of this platform. The data were analyzed according to the processes listed in Fig. 1.

### Identification of lncRNAs associated with the Hippo signaling pathway

A total of 22 Hippo signaling pathway-associated genes (*DCHS1/-2*, *FAT1/-2/-3/-4*, *FRMD6*, *LATS1/-2*, *MST1*, *NF2*, *TAZ*, *TEAD1/-2/-3/-4*, *WWC1*, *MOBKL1A*, *MOBKL1B*, *SAV1*, *MST2*, and *YAP*) were extracted from a previous study^18^. To explore the key lncRNAs associated with the Hippo signaling pathway, Pearson correlations of these 22 genes with lncRNA expression were investigated using the cor() function in R (version 3.6.1). A correlation coefficient of > 0.4 or < − 0.4, as well as *P* < 0.05, were selected as the significance thresholds for screening the Hippo pathway associated lncRNAs.

### The prognostic lncRNAs investigation

Prognostic lncRNAs were screened from the Hippo pathway associated lncRNAs using univariate Cox regression provided in the survival package (version:2.41-1) in R^19^, and *P* < 0.05 was considered as statistical significance.

### Unsupervised cluster analysis

Unsupervised cluster analysis was conducted to determine the different molecular subtypes based on the expression of Hippo pathway-related prognostic lncRNAs. The pheatmap (version:1.0.8)^20^ in R was used to conduct centered Pearson correlation-based bidirectional hierarchical clustering. Differences in overall survival between the different molecular subtypes were assessed using the Kaplan–Meier (KM) curve. Differences in clinical information were also compared between different molecular subtypes.

### Immune infiltration and checkpoint investigation between subtypes

CIBERSORT is useful computational method for quantifying fractions of diverse cell types from bulk tissue gene expression profiles. CIBERSORT can accurately infer the immune composition of tumor tissue by integrating linear support vector regression with known expression profiles of immune cell subsets^21^. To explore the characteristics of immune infiltration in LIHC, CIBERSORT was used to calculate the proportion of immune cells based on the expression profiles TCGA tumor samples, and the differences in terms of immune cells fractions between molecular subtypes were compared. Based on the expression levels in TCGA dataset, a total of 14 specific immune checkpoint genes were extracted, followed by gene expression comparison between different molecular subtypes.

### Prognostic model construction

Associations between Hippo pathway-related prognostic lncRNAs and clinical survival information of samples were further investigated using multivariate Cox regression provided in the R survival package (version2.41-1)^19^. *P* < 0.05 was used as the cut-off value for independent prognostic lncRNA exploration. LASSO is an approach for selecting and shrinking variable in Cox proportional hazards model, which decreases the estimated variance while giving an interpretable final model. LASSO^22^ in the R penalized package (version:0.9.50)^23^ was used to identify the most valuable independent prognostic lncRNAs. The cross-validation likelihood (CVL) algorithm was used to obtain the optimized parameter lambda in the screening model through 1000 computation cycles. Based on the most valuable independent prognostic lncRNAs, a prognosis risk model was developed, from which the risk score was calculated: prognostic score (PS) = ∑Coef _lncRNAs_ × Exp _lncRNAs_ (Coef _lncRNAs_: the prognostic coefficient of lncRNAs in multivariate Cox regression analysis; Exp _lncRNAs_: the expression level of lncRNAs).

### Prognostic model evaluation

The tumor samples from TCGA and the E-TABM-36 (validation set) datasets were randomly assigned to two risk groups based on their median values. The K-M method in the survival package (version 2.41-1) in R^19^ was used to determine the correlation between the risk group and actual survival. The pROC package (version:1.12.1) in R was used to compute the sensitivity and specificity of the ROC curves and reveal the effect of the prognostic risk model. Independent prognostic clinical factors in TCGA dataset were investigated using regression analysis^19^. *P* < 0.05 was set as the cut-off value for the current selection. Nomograms are widely applied for evaluate prognosis of patients in tumor treatments, which can reduce statistical prediction models to a single numerical estimate of the probability of an event, tailored to a single patient's situation, so as to provide clinical decision making in clinical^24^. The identified independent prognostic factors were used to establish nomogram for predicting 3- and 5-year survival using the rms package (version:5.1-2)^25^ in R.

### Genes expression and pathways between risk groups

The limma package (version:3.34.7)^26^ in R was used to identify the differentially expressed genes (DEGs) within the two risk groups with false discovery rate (FDR) < 0.05 and |log_2_FC|> 0.5. Gene set enrichment analysis (GSEA) is an analytical method for evaluating genome-wide expression data at the level of priori defined gene sets, which can be used to determine the gene set that associated with the phenotypic classification^27^. In this study, the altered KEGG pathways between risk groups were investigated using GSEA, with the the priori defined KEGG gene sets, and adjusted *P* < 0.05 was used as the significance cut-off value.

## Results

### Hippo signaling pathway-associated lncRNA investigation

In total, 2528 lncRNAs and 18,497 mRNAs were identified in TCGA dataset after reannotation. After Pearson correlation analysis between genes in the Hippo signaling pathway and the annotated lncRNAs, 313 Hippo signaling pathway-associated lncRNAs and 799 gene-lncRNA co-expression pairs were investigated, including 82 negative and 717 positive correlated pairs (Supplementary Table 1). These 313 lncRNAs were used in subsequent analyses according to the analysis processes showed in Fig. 1.

### Prognostic lncRNAs and unsupervised cluster analysis

**Figure 2:**
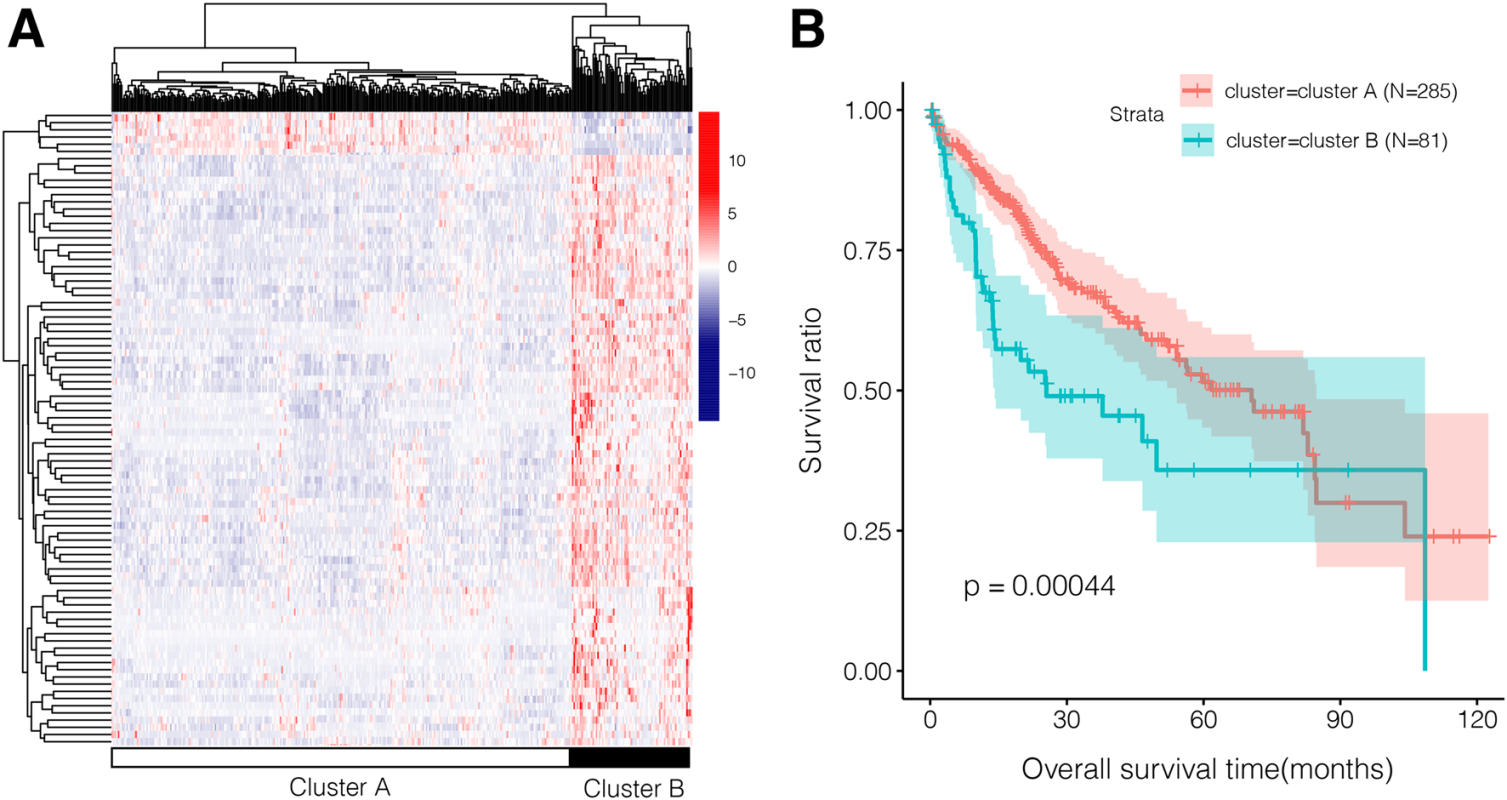
Clustering heatmap and survival analysis. (**A**) Clustering heatmap for Hippo pathway-associated prognostic lncRNAs; red and blue marks represent upregulated and downregulated genes, respectively. (**B**) Kaplan–Meier (KM) survival analysis based on two clusters assembled by lncRNAs; the green marks represent cluster B, while the red marks represent cluster A.

Univariate Cox regression analysis revealed 88 prognostic Hippo pathway-related lncRNAs, and two molecular subtypes were identified based on the expression of these lncRNAs (Fig. 2A). Most samples (n = 285) were clustered into cluster A, and 80 samples were clustered into cluster B. The samples in cluster A had longer survival times than those in cluster B (Fig. 2B). Additionally, these two subtypes differed markedly in terms of clinical characteristics, including age, sex, pathologic T, pathologic stage, histologic grade, vascular invasion, and death (Table 1). For example, the cluster A subtype had relatively more patients with pathologic T1 (55.79% vs. 25.93%), pathologic stage I (52.63% vs. 24.69%), and non-vascular invasive tumors (60.35% vs. 40.74%) than the cluster B subtype.

**Table 1:** Comparison of clinical information between two clusters.

Clinical characteristics	Cluster A (N = 285)	Cluster B (N = 81)	*P* value
Age (years, mean ± sd)	60.31 ± 13.53	57.38 ± 12.48	5.453E−03
Gender (Male/Female)	201/84	46/35	2.264E−02
Pathologic M (M0/M1/–)	199/3/83	64/0/17	9.963E−01
Pathologic N (N0/N1/–)	190/2/93	58/2/21	2.408E−01
Pathologic T (T1/T2/T3/T4/–)	159/62/50/11/3	21/30/28/2/–	5.496E−06
Pathologic stage (I/II/III/IV/–)	150/58/54/4/19	20/27/29/0/0	1.308E−05
Histologic grade (G1/G2/G3/G4/–)	50/144/80/7/4	5/32/38/5/1	6.390E−04
Vascular invasion (Yes/No/–)	77/172/36	30/33/18	1.707E−02
Recurrence (yes/no/–)	105/144/36	35/35/11	2.762E−01
Death (dead/alive)	92/193	38/43	1.793E−02
Overall survival time (months, mean ± sd)	29.01 ± 24.66	20.96 ± 22.06	7.515E−03

*sd* standard deviation.

### Tumor-infiltrating immune cells and immune checkpoint between subtypes

**Figure 3:**
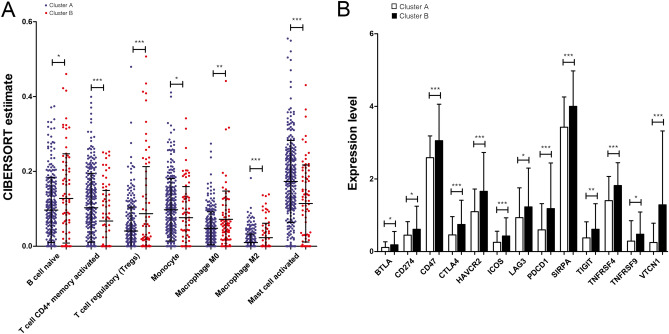
Analysis of immune cells between clusters A and B. (**A**) Immune cells differentially expressed between two clusters. The x-axis shows different types of immune cells, while the y-axis shows the CIBERSORT estimate; the red node represents samples in cluster B, while the blue node represents samples in cluster A. (**B**) Immune checkpoint gene expression analysis between the two clusters; the x-axis represents different immune checkpoint genes, while the y-axis represents the expression level of certain genes.

A total of 22 types of immune cells were analyzed using TCGA dataset. The infiltrating abundances of the seven immune cells showed significant differences between the two subtypes (Fig. 3A). The abundance of tumor-infiltrating activated memory CD4^+^ T cells, monocyte cells, and activated mast cells in cluster A was significantly higher (all *P* < 0.05) than that in cluster B. The abundance of naive B cells, regulatory T (Treg) cells, and M0 and M2 macrophages in cluster B was elevated (all *P* < 0.05) compared to that in cluster A. Furthermore, multiple checkpoint genes were aberrantly expressed between the two subtypes (Fig. 3B). For example, CTLA-4 and PD1/PD-L1 were highly expressed in cluster B than in cluster A (all *P* < 0.05).

### Prognostic model construction

**Figure 4:**
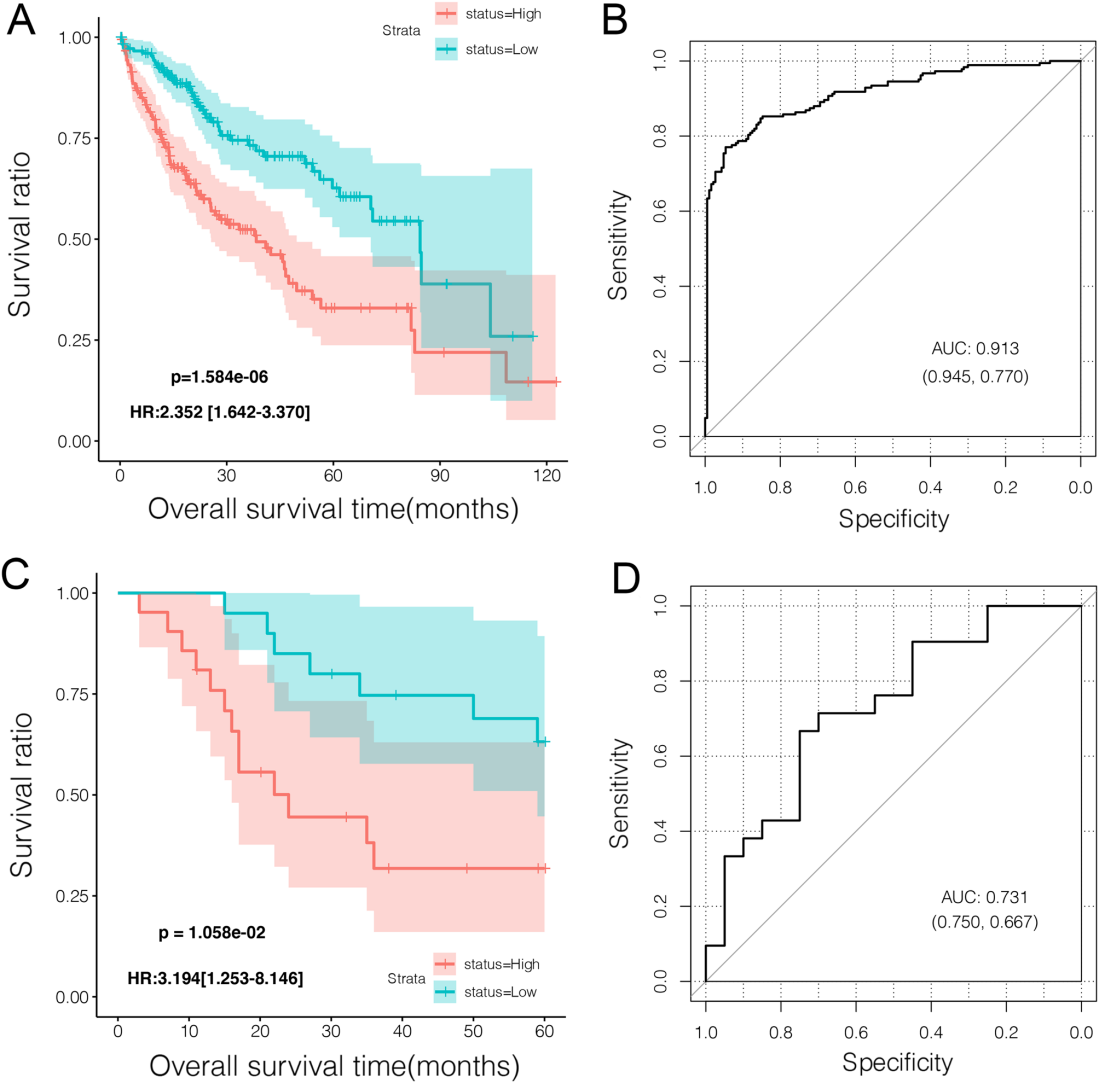
Effectiveness evaluation of the risk prediction model constructed by lncRNAs in the current study. (**A**) Kaplan–Meier survival analysis for the risk model based on clinical data in TCGA dataset; the blue and red lines represent the low- and high-risk groups, respectively. (**B**) Results of ROC analysis on the risk model based on TCGA dataset. (**C**) Kaplan–Meier survival analysis for the risk model based on clinical data in the E-TABM-36 dataset; the blue and red lines represent the low- and high-risk groups, respectively. (**D**) Results of ROC analysis of the risk model based on the E-TABM-36 dataset.

Multivariate Cox regression analysis identified 12 independent prognostic lncRNAs. LASSO regression was used to explore the six most valuable independent prognostic lncRNAs: JMJD1C-AS1, LINC01410, LINC01503, RBM5-AS1, RHPN1-AS1, and TMEM220-AS1 (Table 2). Based on these six lncRNAs, construction of the PS model and calculation of the risk score were performed, followed by risk grouping. In TCGA dataset, patients in the high-risk group showed poorer outcomes than those in the low-risk group (Fig. 4A). Meanwhile, ROC analysis indicated an AUC of 0.913 with a specificity of 94.5% and a sensitivity of 77.0%, indicating a good prognostic effect on the current PS model (Fig. 4B). Comparable results were observed in the E-TABM-36 validation dataset, in which high-risk patients had a short survival time, an AUC of 0.731, a specificity of 75.0%, and a sensitivity of 66.7% (Fig. 4C,D).

**Table 2:** The optimal lncRNA combination in current study.

Symbol	Multi-variable cox regression			LASSO coef
	Hazard ratio	95% CI	Pr( >|z|)	
JMJD1C-AS1	1.581	1.082–1.840	1.368E−02	0.21822
LINC01410	1.941	1.010–2.186	2.850E−02	0.40323
LINC01503	1.329	1.093–1.726	3.567E−02	0.04857
RBM5-AS1	0.729	0.572–0.932	1.160E−02	-0.36305
RHPN1-AS1	1.057	1.012–1.104	1.290E−02	0.47526
TMEM220-AS1	0.966	0.936–0.998	3.790E−02	-0.42233

*CI* confidence interval, *LASSO*,least absolute shrinkage and selection operator.

### Risk score has an independent prognostic value

**Figure 5:**
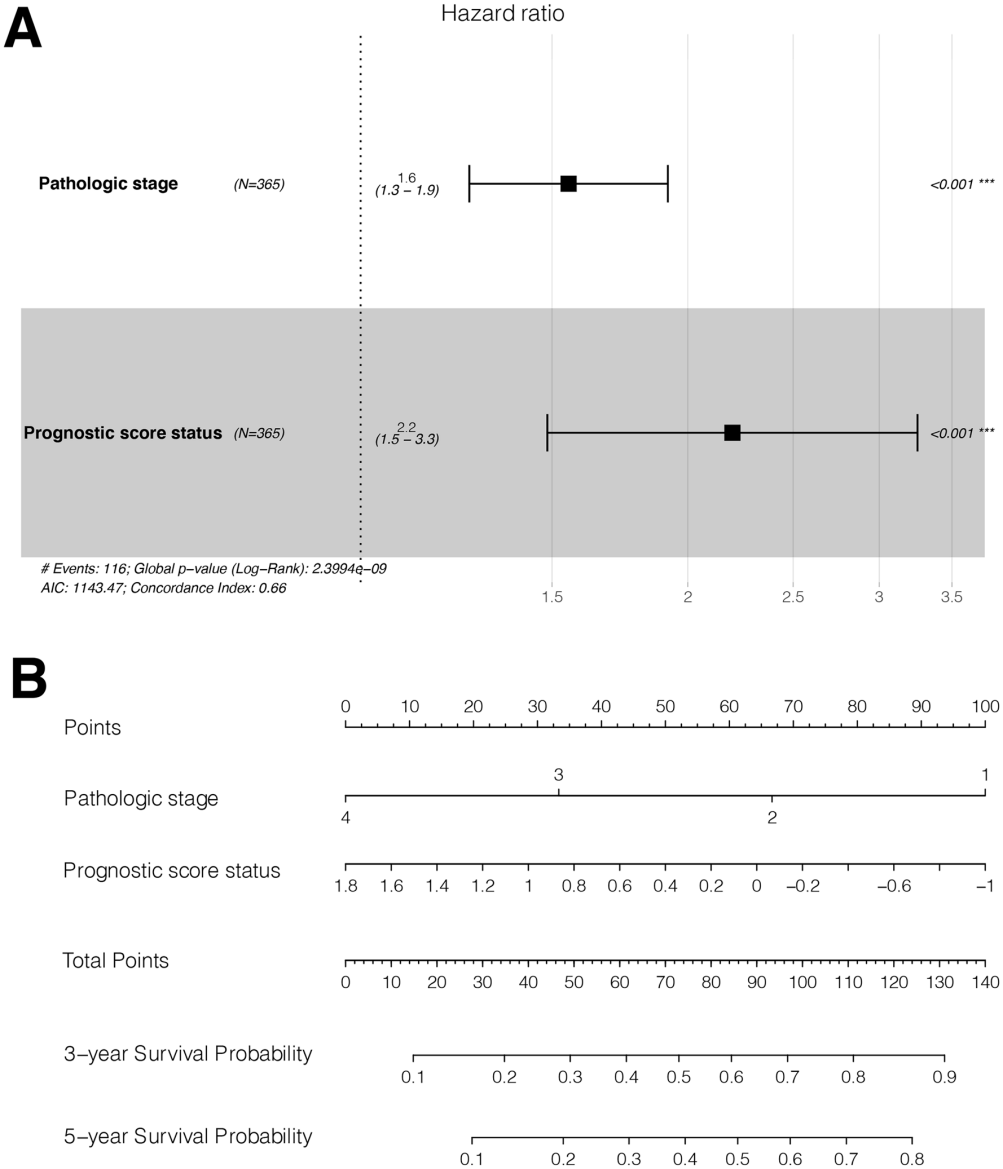
Construction for prognostic Nomogram (**A**) Forest plot shows the two independent prognostic factors in multivariate Cox regression analysis. (**B**) Nomogram developed based on independent prognostic factors.

Cox regression analysis identified two independent prognostic factors: pathologic stage (HR = 1.661, 95% CI 1.355–2.037, *P* < 0.01) and performance status (high or low) (HR = 2.327, 95% CI 1.624–3.333, *P* < 0.01) (Fig. 5A and Table 3). These two independent prognostic factors were included in the nomogram, suggesting that the nomogram had better predictive power for the 3- and 5-years survival probabilities for LIHC patients (Fig. 5B).

**Table 3:** The independent prognostic factors revealed by univariate and multivariate Cox regression.

Clinical characteristics	Uni-variable cox		Multi-variable cox	
	HR (95% CI)	*P* value	HR (95% CI)	*P* value
Age (years, mean ± sd)	1.012 [0.998–1.026]	7.904E−02	–	–
Gender (Male/Female)	0.817 [0.573–1.164]	2.618E−01	–	–
Pathologic M (M0/M1/–)	4.032 [0.978–12.83]	5.198E−02	–	–
Pathologic N (N0/N1/–)	2.004 [0.491–8.181]	3.327E−01	–	–
Pathologic T (T1/T2/T3/T4/–)	1.675 [0.897–2.007]	1.017E−01	–	–
Pathologic stage (I/II/III/IV/–)	1.661 [1.355–2.037]	1.034E−06	1.554 [1.259–1.917]	4.030E−05
Histologic grade (G1/G2/G3/G4)	1.121 [0.887–1.416]	3.392E−01	–	–
Vascular invasion (Yes/No/-)	1.351 [0.892–2.047]	1.537E−01	–	–
Recurrence (Yes/No/–)	1.375 [0.914–2.068]	1.249E−01	–	–
PS status (High/Low)	2.327 [1.624–3.333]	2.163E−06	2.198 [1.485–3.253]	8.240E−05

*CI* confidence interval, *HR* hazard ratio, *PS* prognostic score.

### DEGs between risk groups

**Figure 6:**
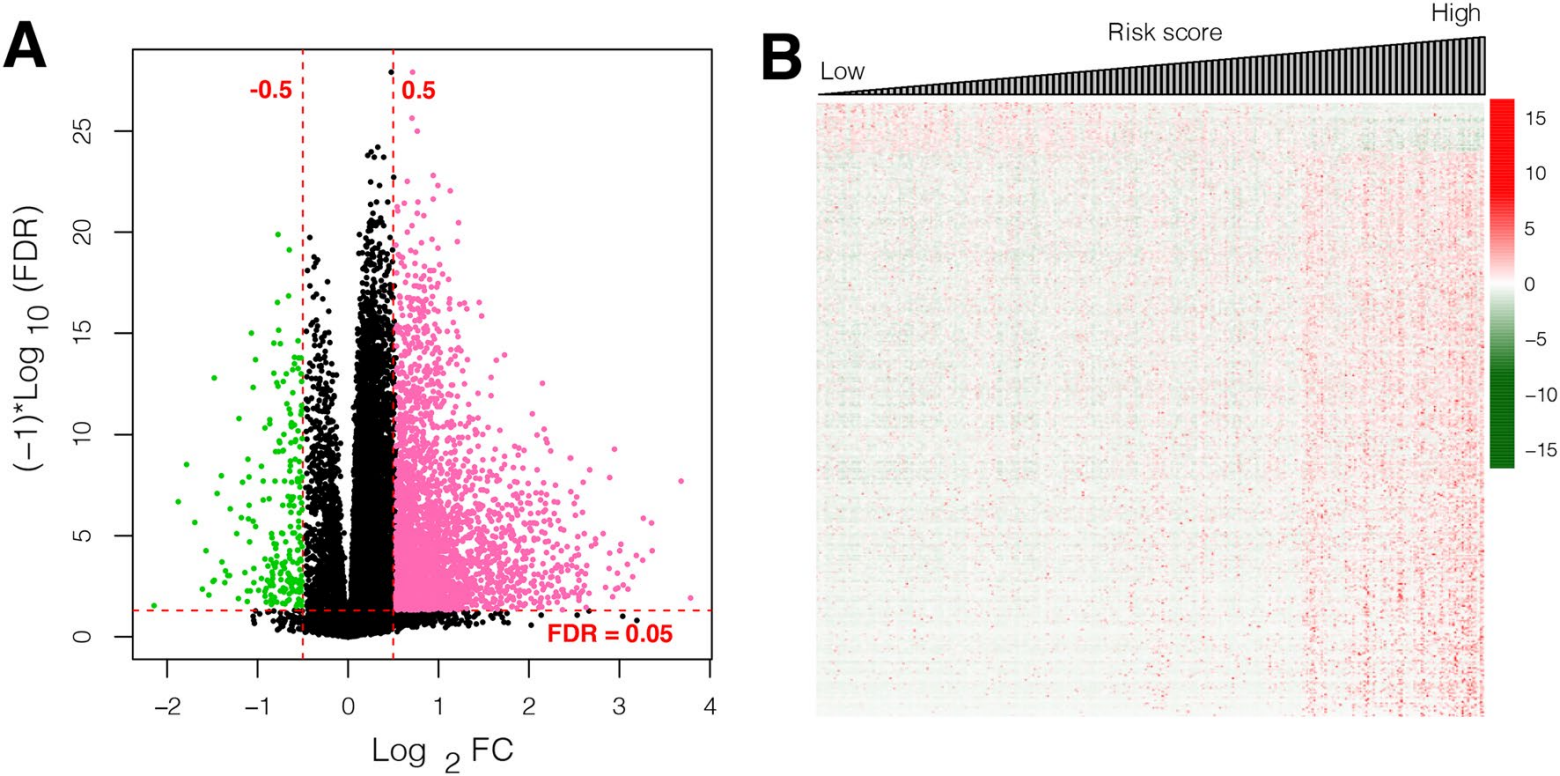
Gene expression between two risk groups (**A**) Volcano plot of gene expression; pink and green nodes show the upregulated and downregulated genes, respectively; horizontal and vertical lines represent FDR < 0.05 and |log_2_FC|> 0.5, respectively. (**B**) Heatmap for dysregulated genes between the two risk groups: red and green marks represent upregulated and downregulated genes, respectively.

A total of 3007 DEGs were detected within the two risk groups, of which the majority were overexpressed in the high-risk group (Fig. 6A). The expression pattern changed from low to high, according to the risk score (Fig. 6B). GSEA enrichment analysis revealed seven significantly enriched KEGG pathways (Supplementary Table 2). Five pathways, including the drug metabolism cytochrome P450 pathway, were markedly implicated in the low-risk group. Meanwhile, two pathways, including the cell cycle and pathway in cancer, were significantly associated with the high-risk group (Supplementary Fig. 1).

## Discussion

Although the Hippo pathway and associated genes are vital for the development of LIHC^6,28^, the prognostic role and molecular mechanisms of Hippo pathway-related lncRNAs in LIHC progression remain unclear. The results of previous studies suggest that the mining of multi-data molecular subtypes is the basis for personalized clinical interventions in cancer^12,29^. Cancer subtypes differ in clinical factors, such as overall survival time, which is often an important indicator of personalized therapy^30^. In this study, according to the expression of 88 Hippo pathway-related prognostic lncRNAs, two molecular subtypes (clusters A and B) were identified. Patients in cluster A had longer survival than those in cluster B; therefore, the clinical characteristics of these two molecular subtypes were compared to investigate possible reasons for this difference. We found that the cluster A subtype had relatively more patients with pathologic T1 (55.79% vs. 25.93%), stage I (52.63% vs. 24.69%), and non-vascular invasive tumors (60.35% vs. 40.74%). This might explain the favorable prognosis of patients with the cluster A subtype.

Additionally, the immune response is closely related to the molecular mechanisms of cancer subtypes^31,32^. We found that the infiltrating abundance of the seven immune cells was significantly different between subtypes A and B. For example, the abundance of tumor-infiltrating activated memory CD4^+^ T cells was elevated in cluster A, while the abundance of Tregs and M2 macrophages was significantly higher in cluster B. Various antitumor biological effects of CD4^+^ T cells have been revealed in a previous study^33^. Activation of CD4^+^ T cells caused by tumor invasion can inhibit inflammation, which is used in the prognosis of head and neck squamous cell carcinoma^34^. Tumor cells have been demonstrated to play a direct role in the expansion of CD4^+^ T cells, thus further inhibiting antitumor immunity due to T cells^35^. Accumulating evidence suggests that Treg cells play a vital role in inhibiting natural killer cell immune responses in human cancer^36^. Yu et al.^37^ showed that tumors with an increased number of Treg cells were associated with poor prognosis in LIHC. A previous study indicated that Treg cells foster tumor progression and predict adverse outcomes in LIHC^38^. Moreover, M2-derived exosomes can accelerate the migration and invasion of colon cancer cells^39^. CHI3L1 proteins produced by tumor M2 macrophages play crucial roles in the progression of tumors in humans^40^. Furthermore, 14 immune checkpoint genes, including *CTLA-4* and *PD1/PD-L1*, were significantly upregulated in cluster B. As an inhibitory co-receptor, CTLA-4 not only interferes with the activation of T cells but is also overexpressed in patients with LIHC^41^. Checkpoint antibody inhibitors, including PD1 and PD-L1 inhibitors, are also common inhibitors with certain tumor-suppressive effects^42^. These findings further explain the differences in the overall survival of patients between the two subtypes.

Among the 88 Hippo pathway-related prognostic lncRNAs, 12 independent prognostic lncRNAs were identified, and LASSO regression identified six valuable independent prognostic lncRNAs, including JMJD1C-AS1, LINC01410, LINC01503, RBM5-AS1, RHPN1-AS1, and TMEM220-AS1. A recent study showed that RHPN1-AS1 promotes malignant progression and predicts poor clinical outcomes in liver cancer^43^. It has also been found to promote tumor cell progression in human cancer by participating in the miR-299-3p/FGF2 axis^44^. A previous study showed that LINC01410 promotes angiogenesis and metastasis in human cancers^45^. A recent study indicated that upregulated LINC01410 presents a poor prognosis in cholangiocarcinoma patients and can be used as a prognostic gene for cancer^46^. Wang et al.^47^ showed that upregulated LINC01503 contributes to cancer cell progression through the MAPK/ERK pathway, which is considered a therapeutic target for LIHC. RBM5-AS1 is involved in the realization of related molecular functions in colon CSCs^48^. Du et al.^49^ indicated that the downregulation of TMEM220-AS1 with copy deletion was associated with a poor prognosis of LIHC, which could be used as a promising prognostic biomarker. These studies suggest the important roles of these lncRNAs. Based on these six lncRNAs, a prognosis risk model was developed to assign patients into two risk -groups, in which patients in the high-risk group had shorter survival times than low-risk patients. Additionally, the risk score was found to have an independent prognostic value in patients with LIHC. This further confirms the prognostic value of these lncRNAs.

Genes that were dysregulated between the two risk groups were also analyzed. Most gene expression levels were elevated in the high-risk group. GSEA revealed that pathways in cancer and the cell cycle were significantly associated with the high-risk group. Under normal circumstances, the damage to endogenous and exogenous DNA generated during cancer development can be repaired by the cell cycle pathway^50,51^. A previous study showed that oncogenic H2AZ1 plays an established role in accelerating the cell cycle transition during hepatocarcinogenesis^52^. In addition, the pathway in cancer is meaningful because it helps reverse, delay, or prevent the occurrence of tumors from a therapeutic point of view^53^. Our study showed that cancer and cell cycle pathways were significantly related to the high-risk group, indicating that the genes in this group contributed to the progression of LIHC.

To our knowledge, this is the first study to investigate the involvement of Hippo pathway-related lncRNAs in the progression of LIHC. We demonstrated that expression pattern of Hippo pathway-related lncRNAs could stratify LIHC patients into different subtypes, emphasizing the heterogeneity of LIHC. Additionally, a prognostic lncRNA signature linked to Hippo pathway was identified to predict prognosis for LIHC patients. Despite of this, there were also limitations in this study. Six prognostic lncRNAs were preliminarily identified to predict the prognosis of LIHC. However, expression of these lncRNAs were not confirmed in clinical samples, especially in biological fluids samples (like serum), and their prognostic value should also be further confirmed by clinical data. Moreover, the advancements in computational biology make it possible to explore the interacted microRNAs of these lncRNAs^54–56^. Therefore, future work in terms of the biological function of these lncRNAs should be conducted to investigate the role and the underlying molecular regulatory mechanism.

In conclusion, two molecular subtypes of LIHC based on the Hippo pathway-related prognostic lncRNAs were identified in this study. These two subtypes differ in terms of overall survival, clinical pathology, infiltration abundance of 7 immune cells, and expression of checkpoint genes such as *CTLA-4* and *PD-1/L1*. Moreover, a prognostic risk model was developed using six independent prognostic lncRNAs (JMJD1C-AS1, LINC01410, LINC01503, RBM5-AS1, RHPN1-AS1, and TMEM220-AS1). This risk model can independently predict the prognosis of LIHC.

## Supplementary Information

Supplementary Information 1.

Supplementary Information 2.

Supplementary Information 3.

Supplementary Information 4.

Supplementary Information 5.

## Data Availability

The datasets generated and/or analysed during the current study are available in the The Cancer Genome Atlas (TCGA) database (https://www.cancer.gov/) and the European Bioinformatics Institute (EBI) array database (http://www.ebi.ac.uk/).
