# Supplementary material for: Investigation of Hippo pathway-related prognostic lncRNAs and molecular subtypes in liver hepatocellular carcinoma
Source: Sci Rep. 2023 Mar 20;13:4521. doi: 10.1038/s41598-023-31754-x (PMC10027880; doi:10.1038/s41598-023-31754-x)
Supplement: Supplementary file 1 — Supplementary Information 1. [file 41598_2023_31754_MOESM1_ESM.docx]

**Legends for supplementary files**

**Supplementary figure 1.** The significantly enriched pathways in gene set enrichment analysis.

**Supplementary table 1.** Gene-lncRNAs co-expression pairs screened in correlation analysis.

**Supplementary table 2.** The significant KEGG pathways enriched by DEGs in Gene set enrichment analysis.

**Supplementary raw data 1.** Expression matrix of all annotated lncRNAs and mRNAs.

**Supplementary raw data 2.** Expression data all lncRNAs and the genes in of Hippo pathway.

**Supplementary raw data 3.** The identified 88 prognostic-associated lncRNAs in univariate cox regression analysis.

**Supplementary raw data 4**. The detailed information for samples in two clusters.

**Supplementary raw data 5.** Immune infiltration value of 22 immune cells estimated by CIBERSORT.

**Supplementary raw data 6.** The 12 independent prognostic lncRNAs identified in multivariate Cox regression analysis.

**Supplementary raw data 7.** Details for samples in high risk and low risk groups.

**Supplementary raw data 8.** Differentially expressed genes between high risk and low risk groups.
