# Supplementary figures and images for: Investigation of Hippo pathway-related prognostic lncRNAs and molecular subtypes in liver hepatocellular carcinoma
Source: Sci Rep. 2023 Mar 20;13:4521. doi: 10.1038/s41598-023-31754-x (PMC10027880; doi:10.1038/s41598-023-31754-x)

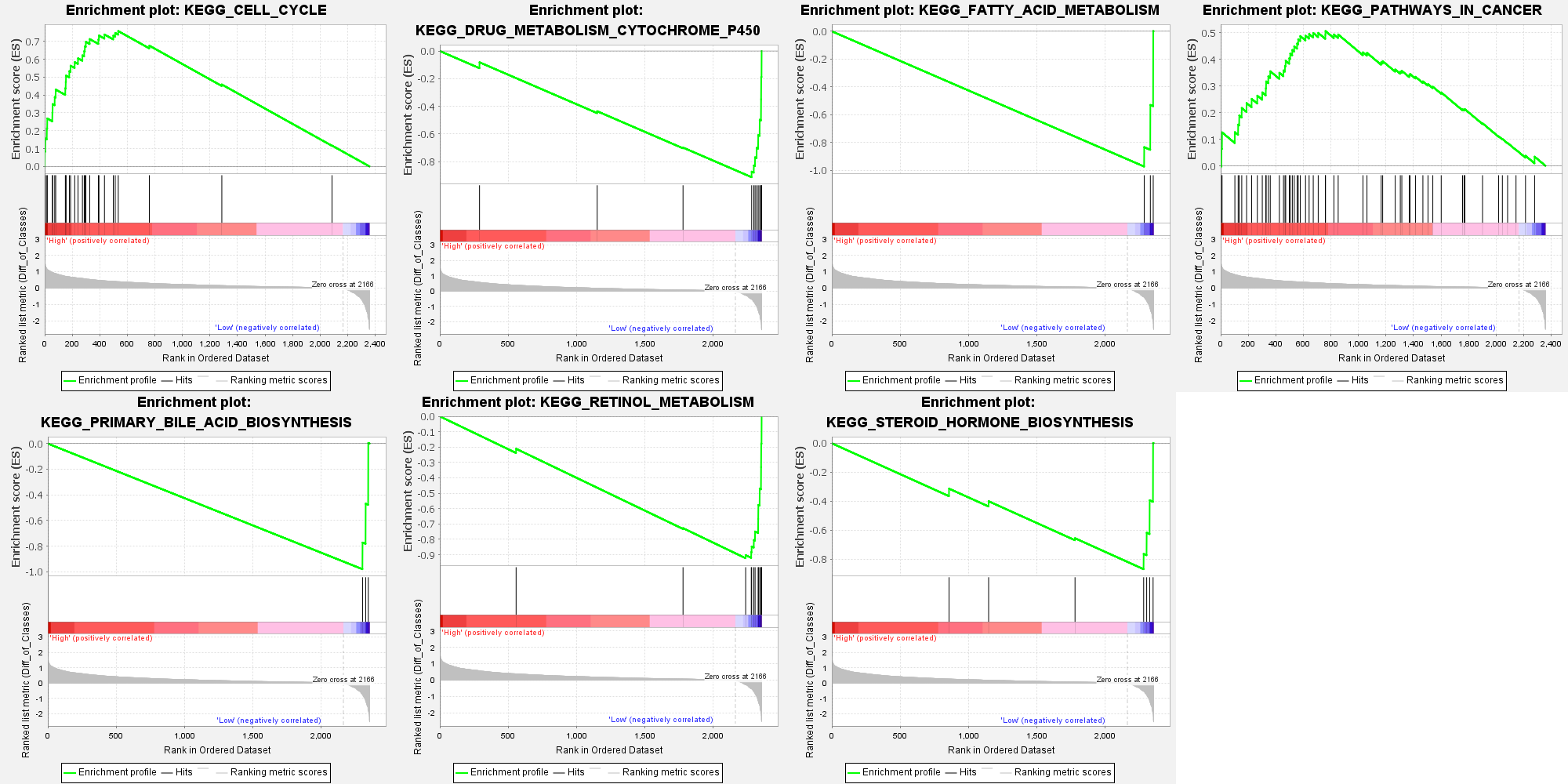

Supplement: Supplementary file 2 — Supplementary Information 2. [file 41598_2023_31754_MOESM2_ESM.tif]
