# Supplementary material for: Investigation of Hippo pathway-related prognostic lncRNAs and molecular subtypes in liver hepatocellular carcinoma
Source: Sci Rep. 2023 Mar 20;13:4521. doi: 10.1038/s41598-023-31754-x (PMC10027880; doi:10.1038/s41598-023-31754-x)
Supplement: Supplementary file 5 — Supplementary Information 5. [file 41598_2023_31754_MOESM5_ESM.docx]

**Supplementary Table 2 The significant KEGG pathways enriched by DEGs**

| NAME | SIZE | ES | NES | P value |
| --- | --- | --- | --- | --- |
| KEGG_DRUG_METABOLISM_CYTOCHROME_P450 | 12 | -0.9119444 | -1.3413932 | 7.879E-03 |
| KEGG_RETINOL_METABOLISM | 13 | -0.9226449 | -1.3207219 | 1.980E-02 |
| KEGG_PRIMARY_BILE_ACID_BIOSYNTHESIS | 3 | -0.9787776 | -1.2086594 | 2.772E-02 |
| KEGG_CELL_CYCLE | 29 | 0.75795 | 1.3418083 | 2.869E-02 |
| KEGG_FATTY_ACID_METABOLISM | 3 | -0.97156197 | -1.2064319 | 3.579E-02 |
| KEGG_STEROID_HORMONE_BIOSYNTHESIS | 7 | -0.8695715 | -1.2735811 | 3.929E-02 |
| KEGG_PATHWAYS_IN_CANCER | 57 | 0.5066864 | 1.2556263 | 4.760E-02 |

**Notes:** KEGG, Kyoto Encyclopedia of Genes and Genomes; DEMs, differentially expressed mRNAs; SIZE, the number of genes Enriched in certain pathway; ES, enrichment score; NES, normalized enrichment score.
